# Coordinated Meta-Storms enables comparison of million-level microbiomes

**DOI:** 10.1093/bioinformatics/btaf438

**Published:** 2025-08-04

**Authors:** Minan Wang, Hao Gao, Yu Zhang, Yongxiang Huang, Xiaoquan Su

**Affiliations:** College of Computer Science and Technology, Qingdao University, Qingdao, Shandong 266071, China; College of Computer Science and Technology, Qingdao University, Qingdao, Shandong 266071, China; College of Computer Science and Technology, Qingdao University, Qingdao, Shandong 266071, China; College of Computer Science and Technology, Qingdao University, Qingdao, Shandong 266071, China; College of Computer Science and Technology, Qingdao University, Qingdao, Shandong 266071, China

## Abstract

**Motivation:**

Phylogeny-based distance serves as the foundation in the evaluation of microbiome beta diversity. However, the exponential growth of data volume has recently introduced substantial computational challenges, while current methods remain limited in their scalability and efficiency.

**Results:**

To solve these issues, we introduce Coordinated Meta-Storms (CMS), a novel approach for large-scale microbiome phylogeny-based distance calculation. CMS incorporates a dynamic multiple Graphics Processing Unit architecture based on a self-adaptive data decomposition strategy and optimization, which empowers pairwise comparison of million-level microbiomes within 40 h on a single computing node.

**Availability and implementation:**

Coordinated Meta-Storms is implemented by CUDA, HIP and C++. Source code is available at GitHub (https://github.com/qdu-bioinfo/Coordinated-Meta-Storms) and has been published on Zenodo (DOI: 10.5281/zenodo.15802999).

## 1 Introduction

The significance of microbial communities in ecosystems has been increasingly recognized, driving substantial and in-depth research efforts. Recently, hundreds of thousands of samples were used for in-depth exploration of the global microbiome transition network (Jing *et al.* 2021a,b) and the global patterns of human gut microbiome (Abdill *et al.* 2025). A central task in all these analyses is beta diversity assessment, which typically depends on pairwise distance matrices. However, traditional metrics such as Bray-Curtis and Jaccard often fail to accurately capture microbial relationships due to high dimensionality, feature sparsity, and taxonomic misalignment. Phylogeny-based methods, such as UniFrac (Lozupone and Knight 2005) and Meta-Storms (MS) (Su *et al.* 2012), achieve more accurate and comprehensive results, but are computationally intensive and struggle with scalability on ultra-large datasets.

While CPUs are commonly used for general-purpose computing, Graphics Processing Units (GPUs) and GPU-like accelerators (GLAs) provide high parallelism through many-core architectures, making them well-suited for computationally intensive tasks. As a result, many microbiome distance metric methods have adopted GPU-based optimizations to improve efficiency. For instance, UniFrac has been accelerated using OpenACC (Sfiligoi *et al.* 2022), GPU Meta-Storms (GMS) (Su *et al.* 2014) uses CUDA. Despite these advancements, current GPU-accelerated solutions still face significant limitations. First, when processing ultra-large datasets (e.g. over 50 000 samples), the limited memory capacity of GPUs can lead to resource allocation failures. Second, many of these implementations rely solely on a single GPU, failing to fully exploit the capabilities of multi-GPU systems.

## 2 Methods

To address these issues, we propose Coordinated Meta-Storms (CMS), a software package for large-scale microbiome distance calculation utilizing multiple GPUs in a coordinated manner. Building on the MS algorithm, CMS not only optimizes its computing kernel, but also introduces a self-adaptive data decomposition strategy and a multi-GPU coordinate architecture.

### 2.1 Computational optimization of the Meta-Storms algorithm

**Figure 1.**
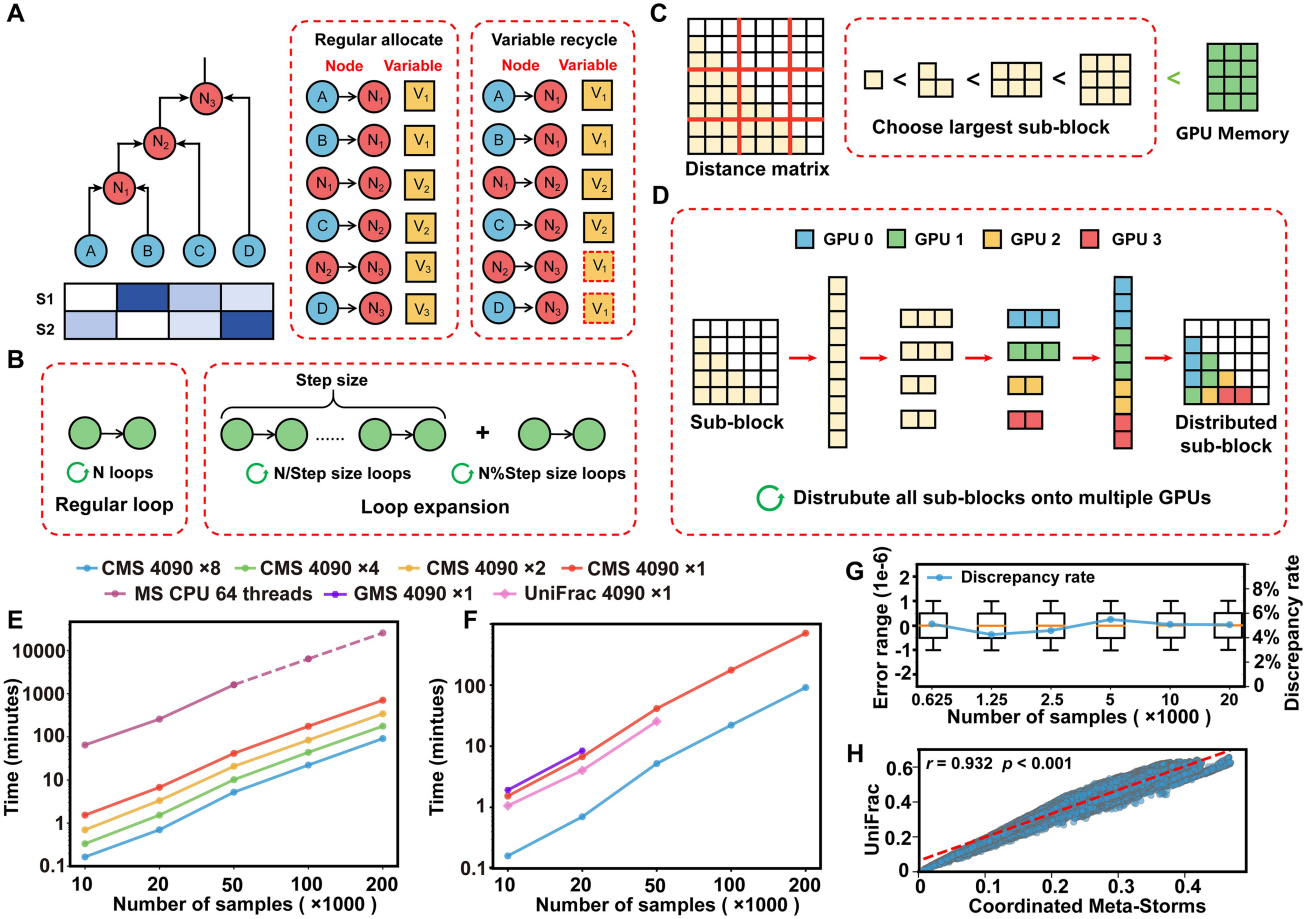
Architecture and performance of CMS. (A) The recursive traversal of the phylogenetic tree (left) is transformed into a non-recursive post-order sequential traversal (middle). For internal nodes (e.g. N1 stored in variable V1), memory is reused after their parent nodes are processed (e.g. V1 reused for N3; right). (B) The individual traversal loops (left) are unified into a fixed step size (right), reducing loop control overhead and avoiding memory access conflicts. (C) The distance matrix computation is decomposed into sub-blocks based on available GPU memory, preventing memory overflow. (D) Sub-block tasks are distributed evenly across multiple GPUs for parallel computation. (E) Total computation time for distance matrix generation using multi-GPU CMS and original MS. Dashed lines indicate extrapolated CPU estimates based on linear regression from smaller datasets. (F) Runtime comparison of GPU-based phylogenetic distance methods. GMS and UniFrac failed to process large matrices due to GPU memory limitations when using a single RTX 4090. (G) Discrepancy range (boxplot) and mean error rate of CMS compared to the original MS implementation. (H) Spearman correlation between distances computed by CMS and UniFrac. The diagonal dashed line indicates the fitted linear regression.

To adapt this algorithm for GPU stream processors, CMS replaces the recursive traversal with a non-recursive thread-based loop (i.e. the kernel function) (Fig. 1A). Moreover, to minimize overhead of loop control and avoid memory access conflicts, we redesigned the kernel function by loop expansion (Fig. 1B). Specifically, the threaded computation traversal loop is expanded, merging iterations with a fixed step size, with the remaining steps processed separately (Fig. S1, available as supplementary data at *Bioinformatics* online).

### 2.2 Self-adaptive data decomposition for large-scale distance matrices

When performing distance calculation, GPU memory usage in this context is primarily determined by the number of samples (matrix dimensions) and the number of microbiome features (leaf nodes in the phylogenetic tree). Due to the limited and non-expandable nature of GPU memory, large microbiome distance matrices cannot be loaded in their entirety for computation. To address this issue, CMS uses a flexible data decomposition strategy that partitions the distance matrix into smaller sub-blocks. Before computation, CMS estimates memory requirements using three strategies: direct comparison, binary decomposition, and quantitative reduction (Fig. 1C; details in Methods, available as supplementary data at *Bioinformatics* online and Fig. S2, available as supplementary data at *Bioinformatics* online). These strategies are applied sequentially to decompose the matrix, ensuring adherence to memory constraints. Once a strategy yields sub-block size that satisfies the GPU memory constraints, CMS bypasses subsequent strategies and initializes a structure to record the positions of these sub-blocks. Pre-sorted microbial features, the corresponding phylogenetic tree, and temporary arrays are then loaded into GPU memory. Each sub-block is processed sequentially, with results merged into the full distance matrix according to the predefined layout.

### 2.3 Coordinated calculation across multiple GPUs

Multi-GPU collaboration is the key to enabling ultra-large scale microbiome analysis. To maximize resource utilization, CMS adopts an intra-block parallelization strategy, distributing computing tasks evenly across available GPUs, avoiding performance bottlenecks caused by an imbalance numbers of sub-blocks and GPUs (Fig. 1D). The computational task queue assigned to each GPU is independently managed. Once the computations are complete, the CPU merges the results from each GPU and frees up GPU resources.

## 3 Results

### 3.1 Datasets and testing environment

To assess CMS’s performance, we randomly selected 200 000 16S amplicon microbiomes from the Microbiome Search Engine (Jing *et al.* 2021a,b). These samples, referred to as the MSE dataset, were annotated using the Greengenes 13–8 database (DeSantis *et al.* 2006). We also downloaded additional 10 000 samples from NCBI and annotated by different reference databases as NCBI dataset (Table S1, available as supplementary data at *Bioinformatics* online; details in Results, available as supplementary data at *Bioinformatics* online). Experiments were conducted on a server equipped with a Xeon Gold 6142 CPU, eight NVIDIA RTX 4090 GPUs and 512GB RAM.

### 3.2 Running speed and parallelization efficiency

To benchmark CMS, we compared its performance against the MS in calculating the MSE dataset. Using 8 RTX 4090 GPUs, CMS completed the distance matrix calculation for 200 000 microbiome samples in just 1.5 h (Fig. 1E), achieving a speedup of 280× over the CPU 64 threads (Fig. S3A, available as supplementary data at *Bioinformatics* online). By comparison, the estimated runtimes for the CPU and single-GPU versions were 428  and 11.8 h, respectively. Meanwhile, the CMS demonstrated excellent parallel efficiency showcasing its effective use of multiple GPUs for enhanced computational performance (Fig. S3B, available as supplementary data at *Bioinformatics* online), while also illustrating significantly reduced memory consumption in ultra-large-scale dataset (Fig. S4, available as supplementary data at *Bioinformatics* online). Given the stable performance trend, CMS was projected to compute the distance matrix for one million samples within 40 h, demonstrating its efficiency even at ultra-large scales. Besides, the HIP (Heterogeneous-computing Interface for Portability) version of CMS on GLAs also performed impressively in calculation speed, speedup, and parallel efficiency aspects (Fig. S5, available as supplementary data at *Bioinformatics* online; details in Results, available as supplementary data at *Bioinformatics* online).

We further assessed the performance of CMS through NCBI dataset to verify the powerful scalability to process microbiomes annotated against other references. While the peak running time varied with database size, the overall speedup trend remained consistent across all cases (Fig. S6, available as supplementary data at *Bioinformatics* online). Specifically, using 8 RTX 4090 GPUs, CMS also completed the distance matrix calculation for NCBI dataset annotated by the Greengenes 13–8, SILVA (Quast *et al.* 2013), and RefSeq (Goldfarb *et al.* 2025) databases in approximately 10 seconds. Even for the largest scale Greengenes2 (McDonald *et al.* 2024) database, the calculation is completed within 40 seconds.

Furthermore, a comparative analysis was conducted between CMS, GMS, and UniFrac (Fig. 1F). When tested with 20 000 samples on a single GPU, all three approaches showed comparable runtime. However, CMS demonstrated a clear speed advantage when utilizing multiple GPUs, benefiting from its parallel architecture. More critically, GMS and UniFrac were unable to process datasets exceeding 50 000 samples due to GPU memory limitations. In contrast, CMS successfully overcame this bottleneck through its self-adaptive data decomposition strategy.

### 3.3 Calculation accuracy and diversity consistency

To validate the computational accuracy and consistency of CMS, we first performed cross-platform comparisons between CMS and the regular MS algorithm using the MSE dataset. The discrepancies across platforms were minimal, with an average discrepancy of 4.6% (i.e. over 95% values in the distance matrix were identical) and an error range within 1e-06 (Fig. 1G). Further validation using the ENV dataset (comprising 344 randomly selected microbiomes from 6 habitats within the MSE dataset; Table S1, available as supplementary data at *Bioinformatics* online) revealed a strong Spearman correlation between CMS and UniFrac (*r *= 0.932, *P *< 0.001; Fig. 1H). In addition, Monte Carlo test on the principal coordinates confirmed consistent beta diversity patterns derived from different methods (Procrustes statistic = 0.969, *P *< 0.001; Fig. S7, available as supplementary data at *Bioinformatics* online), supporting the reliability of CMS in capturing microbiome beta-diversity.

## 4 Conclusion

Coordinated Meta-Storms proposes an innovative high-performance computing framework tailored for multi-GPU architectures, delivering highly rapid processing capabilities. This advancement empowers researchers to conduct comprehensive diversity analyses on microbiome datasets containing hundreds of thousands to millions of samples. With its demonstrated efficiency, scalability, and architectural flexibility, CMS enables the exploration of complex microbial ecosystems at unprecedented resolution and throughput, thereby opening new avenues for large-scale, data-intensive microbiome studies.

## Supplementary Material

btaf438_Supplementary_Data

## Data Availability

The software packages and datasets are available at GitHub (https://github.com/qdu-bioinfo/Coordinated-Meta-Storms).
