# Supplementary material for: Coordinated Meta-Storms enables comparison of million-level microbiomes
Source: Bioinformatics. 2025 Aug 4;41(8):btaf438. doi: 10.1093/bioinformatics/btaf438 (PMC12342987; doi:10.1093/bioinformatics/btaf438)
Supplement: btaf438_Supplementary_Data [file btaf438_supplementary_data.doc]

**Supplementary Information for**

# Coordinated Meta-Storms enables comparison of million-level microbiomes

Minan Wang ^1, $^, Hao Gao ^1, $^, Yu Zhang ^1^, Yongxiang Huang ^1^, Xiaoquan Su ^1^, *

^1^ College of Computer Science and Technology, Qingdao University, Qingdao 266071, Shandong, China

* Correspondence to [suxq@qdu.edu.cn](mailto:suxq@qdu.edu.cn)

^$^ Contributed equally, co-first authors

**Supplementary Methods**

*Meta-Storms phylogeny-based distance*

The Meta-Storms (MS) algorithm (Su, et al., 2012) employs a recursive post traversal of a weighted phylogeny tree to compute the difference of two microbiome samples. Specifically, it first calculates the directly shared abundance of two microbiomes on the tip nodes (e.g., OTU or species), and reduces the non-overlapped component to common parent nodes by the phylogeny branches, then it repeats the calculation until the root node of the tree.

*Brief introduction to non-recursive transformation and memory recycling*

The non-recursive transformation and memory recycling strategy was originally developed for GPU-based Meta-Storms (GMS) that we previously developed (Su, et al., 2014) and implemented in CUDA. The recursive traversal of the tree would reduce the computing efficiency due to stack allocation, especially with a deep phylogeny tree, thus all nodes of the tree are expanded in the post-order to transform the traversal into serial mode. In addition, besides the relative abundance of the tip nodes, the calculation needs extra space to store the temporary abundance for internal nodes. Notably, for any of the internal nodes, the temporary space is free for reuse after its parent has been processed (e.g., in Fig. 1A of the main text, the spaces for node N1 are available after the computation of node N2). By employing such a memory recycling strategy, we have reduced the space for internal nodes from tens of thousands to dozens.

*Determination of sub block size for adaptive matrix decomposition*

Before calculation, CMS evaluates the required GPU memory through sample number (matrix dimension) and the number of microbiome features (leaf nodes in the phylogenetic tree). If the on-system GPU device has enough memory, the entire matrix is calculated directly. This direct computation strategy is efficient when the memory requirements are within the GPU's capacity, as it minimizes the overhead associated with matrix partitioning and subsequent recombination.

However, if the GPU memory is insufficient to accommodate the required computational resources for the entire matrix, CMS will adopt a binary decomposition strategy to split the matrix into sub-blocks. This strategy reduces the matrix dimensions by half, thereby breaking down large microbial community distance matrices into smaller, more manageable sub-blocks. This process significantly reduces the GPU memory footprint of the computational tasks, enabling efficient processing.

When the sub block size is smaller than the predetermined threshold, performing binary reduction again will result in wastage of GPU memory. At this point, CMS switches to a quantitative reduction strategy, reducing the sub-block size by a fixed value when below the threshold. This process iterates until all sub blocks meet the GPU's memory limit, ensuring that computing tasks can be executed without exceeding available memory resources and generating the largest sub block area.

**Supplementary Results**

*Dataset selection for performance evaluation experiments*

For performance evaluation of Coordinated Meta-Storms (CMS), we prepared 3 datasets: MSE dataset (200,000 samples), ENV dataset (344 samples), and NCBI dataset (10,000 samples) from different source (details in **Table S1**). All microbiomes were produced by 16S rRNA amplicon sequencing, and short reads were preprocessed by Parallel-Meta Suite (PMS) (Chen, et al., 2022) using OTU (operational taxonomy units) picking against reference full-length 16S rRNA gene databases (**Table S2**).

*Comparison of system memory consumption*

We conducted a comparative analysis of RAM usage between CMS and original MS. For relatively small datasets, CMS exhibited slightly higher memory usage compared to the MS, but this difference was minimal and did not impact overall performance. Then, as the dataset size increased, the CMS consistently demonstrated superior memory efficiency, largely attributable to its optimized matrix decomposition strategy. For example, when processing a large-scale dataset comprising 200,000 microbiome samples on a server equipped with 512 GB of RAM, MS failed to complete the computation due to an out-of-memory error, with an estimated peak memory requirement exceeding 530 GB. In stark contrast, CMS successfully completed the task, reaching a maximum memory usage of only 278 GB. This substantial reduction in memory demand of nearly 40%, highlights the effectiveness of CMS's memory optimization techniques.

*Assessment of CMS on different computing devices*

In addition to CUDA-based CMS, we evaluated the performance of the HIP (Heterogeneous-computing Interface for Portability) based CMS on a heterogeneous computing platform utilizing multiple GPU-like accelerators (GLAs). This assessment was conducted using up to 100,000 samples from the MSE dataset. Despite the architectural disparity between NVIDIA GPUs and GLAs (e.g., GLAs having only 3,840 processors and 16GB on-board memory), CMS demonstrated substantial acceleration and maintained high parallel efficiency. Specifically, when deployed on four GLAs, the HIP-enabled CMS completed pairwise distance matrix computations for 10,000 microbiome samples in approximately 3 minutes (**Fig. S5A**). This corresponds to a 23× speedup when compared to 64 CPU threads (**Fig. S5B**). Moreover, the system exhibited excellent efficiency. The parallel efficiency scaled nearly linearly with the number of GLAs: configurations using two, three, and four GLAs achieved performance improvements of approximately 2×, 3×, and 4× relative to a single GLA, respectively (**Fig. S5C**). These findings affirm the portability and parallel scalability of CMS across diverse hardware environments, making it a viable solution for large-scale analyses on both GPU and GPU-like accelerators.

**Supplementary Figures**


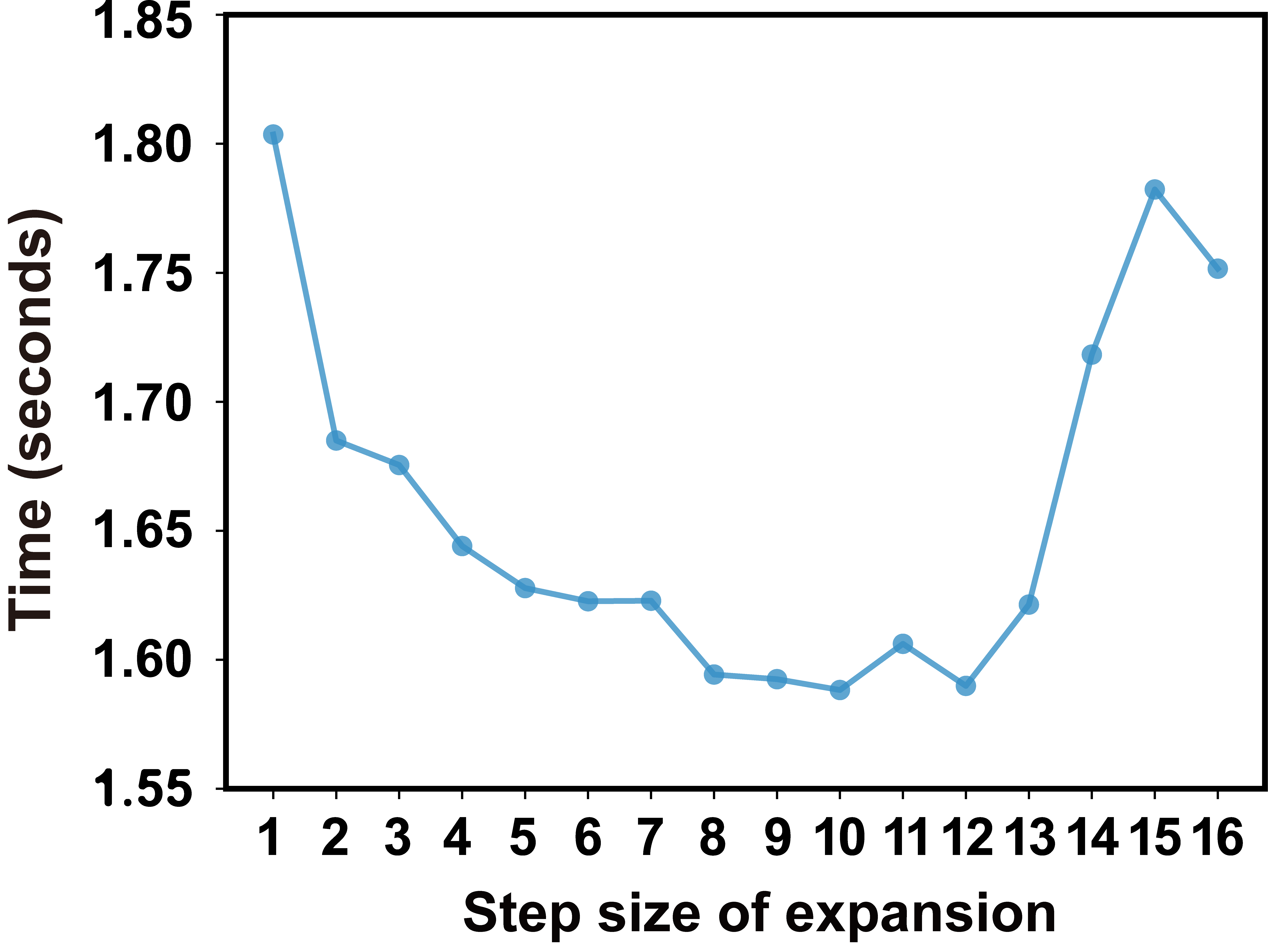


**Fig. S1. Loop expansion test experiment.** The experiment was conducted using a single GLA to calculate the distance matrix. Based on these results, we selected an optimal step size of 10 as the default for subsequent experiments.

**
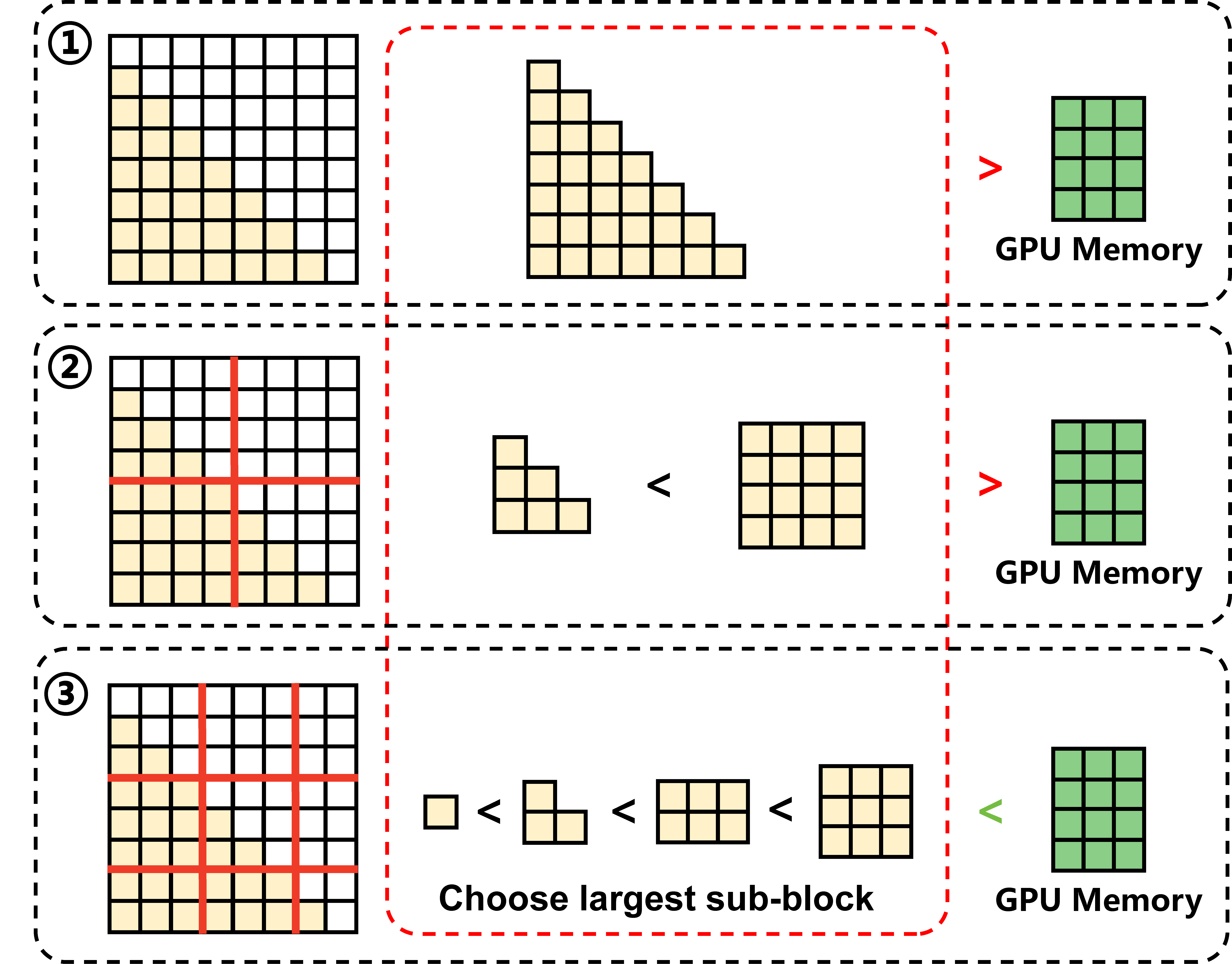
**

**Fig. S2. Self-adaptive data decomposition for large-scale distance matrices.** Steps 1, 2, and 3 respectively demonstrate three decomposition strategies: direct comparison, binary decomposition, and quantitative reduction





**Fig. S3. Performance of speedup (A) and efficiency (B) of CMS compare to MS.**


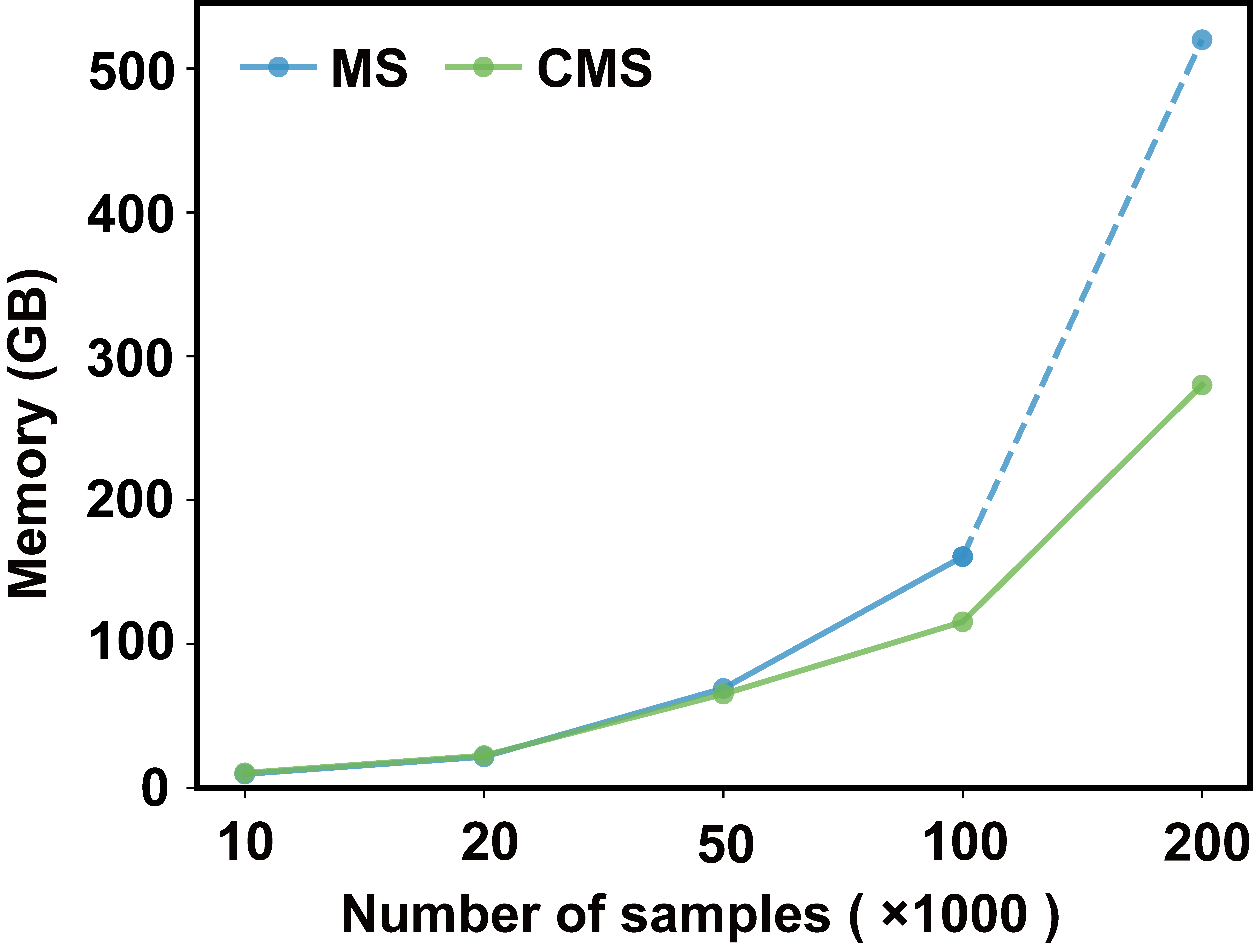


**Fig. S4. Memory consumption of CMS compared to MS**. Dotted lines indicate estimated memory consumption of MS due to RAM insufficient.





**Fig. S5. Performance of HIP version CMS. (A)** Overall calculation time of multiple GLAs CMS and MS. Dotted lines indicate extrapolated results based on linear regression fitted to smaller datasets, due to high computational costs on larger ones. (**B**) Speedup of CMS using multiple GLAs compared to MS using 64 CPU threads. (**C**) Parallel efficiency of HIP version CMS.





**Fig. S6. Performance of CMS with NCBI dataset annotated by different reference databases.** (**A**) Total calculation time for distance matrices of different reference databases. (**B**) Speedup of CMS compared to MS using 64 CPU threads.





**Fig. S7. Principal coordinate analysis of ENV dataset based on (A) CMS and (B) UniFrac distances.** Circles represent the confidence ellipse level of 0.85.

**Supplementary Tables**

**Table S1. Experimental datasets**

| **Dataset** | **# of microbiomes** | **Description** |
| --- | --- | --- |
| MSE dataset | 200,000 | Randomly selected from MSE annotated by Greengenes 13-8 (DeSantis, et al., 2006) |
| NCBI dataset | 10,000 | Randomly selected from studies of PRJEB19825, PRJNA503194, PRJEB14474, and PRJEB11697 annotated by Greengenes 13-8, Greengenes2 (McDonald, et al., 2024), SILVA (Quast, et al., 2013), and RefSeq (Goldfarb, et al., 2024) respectively (details in **Table S2**). |
| ENV dataset | 344 | Randomly selected from gut (50 samples), oral (50 samples), soil (47 samples), marine (60 samples), plant (49 samples) and river (88 samples) environment annotated by Greengenes 13-8. |

**Table S2. Reference 16S rRNA databases**

| **Reference databases** | **# of microbes** |
| --- | --- |
| Greengenes 13-8 | 99,322 |
| Greengenes2 | 331,269 |
| SILVA | 152,264 |
| RefSeq | 101,484 |

**Supplementary References**

Chen, Y.Z.*, et al.* Parallel-Meta Suite: Interactive and rapid microbiome data analysis on multiple platforms. *Imeta* 2022;1(1).

DeSantis, T.Z.*, et al.* Greengenes, a chimera-checked 16S rRNA gene database and workbench compatible with ARB. *Appl Environ Microb* 2006;72(7):5069-5072.

Goldfarb, T.*, et al.* NCBI RefSeq: reference sequence standards through 25 years of curation and annotation. *Nucleic Acids Res* 2024;53(D1):D243-D257.

McDonald, D.*, et al.* Greengenes2 unifies microbial data in a single reference tree (Jul, 10.1038/s41587-023-01845-1, 2023). *Nat Biotechnol* 2024;42(5):813-813.

Quast, C.*, et al.* The SILVA ribosomal RNA gene database project: improved data processing and web-based tools. *Nucleic Acids Res* 2013;41(D1):D590-D596.

Su, X.*, et al.* GPU-Meta-Storms: computing the structure similarities among massive amount of microbial community samples using GPU. *Bioinformatics* 2014;30(7):1031-1033.

Su, X.Q., Xu, J. and Ning, K. Meta-Storms: efficient search for similar microbial communities based on a novel indexing scheme and similarity score for metagenomic data. *Bioinformatics* 2012;28(19):2493-2501.
